# PTBP1-mediated inhibition of circular RNA SCMH1 biogenesis impairs brain recovery after ischemic stroke

**DOI:** 10.7150/thno.114179

**Published:** 2026-01-01

**Authors:** Ying Bai, Bing Han, Yi Zhang, Liying Wen, Chang Liu, Yu Wang, Jiafang Cui, Bingjing Zheng, Ningbo Cai, Lian Xu, Ling Shen, Yuan Zhang, Honghong Yao

**Affiliations:** 1 Department of Pharmacology, Jiangsu Provincial Key Laboratory of Critical Care Medicine, School of Medicine, Southeast University, Nanjing 210009, China.; 2 Southeast University Affiliated Liangjiang Hospital, Nanjing Pukou People's Hospital, Southeast University, Nanjing 211899, China.; 3 Co-innovation Center of Neuroregeneration, Nantong University, Nantong 226001, China.; 4 Institute of Life Sciences, Key Laboratory of Developmental Genes and Human Disease, Southeast University, Nanjing 210096, China.

**Keywords:** ischemic stroke, circSCMH1, astrocyte, PTBP1, biogenesis

## Abstract

Rationale: Aberrant circular RNA (circRNA) expression is implicated in various diseases, but the regulatory mechanisms remain poorly understood. Our previous work identified circSCMH1 as a brain repair-associated circRNA, prompting investigation into its biogenesis regulation.

Methods: We combined computational analysis of RNA-binding protein (RBP) binding sites in flanking intronic regions with transcriptomic sequencing to identify potential circSCMH1 regulators. Molecular biology experiments including RNA immunoprecipitation and functional assays were performed to validate the interaction between candidate RBPs and circSCMH1 precursor sequences.

Results: Polypyrimidine tract binding protein 1 (PTBP1) was identified as a key regulator binding specifically to the 800-882 segment at the 3' end of circSCMH1's flanking intron. This binding event inhibited back-splicing and reduces circSCMH1 production. Functional studies demonstrated that PTBP1-mediated suppression of circSCMH1 exacerbates post-stroke brain injury.

Conclusions: Our study reveals a novel molecular mechanism whereby PTBP1 regulates circSCMH1 biogenesis through suppression of back-splicing. These findings advance understanding of circRNA regulatory networks and suggest potential therapeutic targets for stroke recovery.

## Introduction

Due to the specific enrichment in the central nervous system, circular RNAs (circRNAs) are regarded as important regulatory molecules in brain physiology and pathology^1^. Particularly, circRNAs demonstrate remarkable potential in stroke management by functioning as crucial biomarkers for early detection and outcome prediction, while simultaneously playing pivotal roles in neuroprotection, angiogenesis, and inflammatory response regulation^2^, ^3^. Among these candidates, circular RNA SCMH1 (circSCMH1) has emerged as a particularly promising target, with its efficacy validated through rigorous non-human primate studies^4^. Its cellular and molecular mechanisms that promote brain repair post-stroke have been comprehensively elucidated, including the enhancement of neuronal plasticity^4^, the promotion of vascular repair^5^, and the increase of astrocytic microdomain calcium activities^6^. The compelling results observed in these preclinical models underscore the translational potential of circSCMH1-based therapeutic interventions in addressing the complex challenges of stroke rehabilitation. However, despite these advances, a fundamental scientific question remains unresolved: what mechanisms underlie the observed decrease in circSCMH1 expression following ischemic stroke? Elucidating these regulatory processes remains a critical research priority, potentially shedding light on stroke pathophysiology and revealing novel therapeutic avenues.

RNA biogenesis is characterized by intricate complexity and is governed by a diverse array of regulatory mechanisms. In contrast to linear RNAs, the biogenesis of circRNAs is dependent on a distinctive process known as the precursor mRNAs' back-splicing, which is orchestrated by RNA-binding proteins (RBPs)^7^. Various RBPs, such as heterogeneous nuclear ribonucleoprotein L, fused in sarcoma, and quaking, have been reported to function as *trans*-factors by binding to specific RNA motifs in flanking introns to regulate circRNA biogenesis^7^-^11^. However, circRNAs exhibit distinct specificity across cell types, tissues, developmental stages, and various physiological and pathological conditions^12^, ^13^. Consequently, the regulatory mechanisms governing the generation of different circRNAs are diverse and context-dependent. Despite these developments, current understanding of how RBPs regulate circRNA biogenesis is still not comprehensive. Understanding how specific RBPs interact with their target sequences to precisely regulate circRNA biogenesis in response to diverse cellular environments and environmental cues remains a critical unresolved question.

In this study, we employed the eCLIP database in conjunction with FIMO-based motif scanning to analyze RBP binding sites within flanking intronic regions. By integrating these findings with transcriptomic sequencing data, we identified Polypyrimidine tract binding protein 1 (PTBP1) as a potential regulator of circSCMH1 biogenesis. Mechanistically, we demonstrated that PTBP1 binds to the region (∆800-882) of the downstream flanking intronic sequence of circSCMH1, subsequently inhibiting its reverse splicing. PTBP1 knockdown significantly reversed ischemia-induced decreases in circSCMH1 levels and reduced astrocyte activation markers in functional studies, while* in vivo* experiments using AAV-GFAP-shRNA-*Ptbp1* demonstrated improved functional recovery in stroke models, underscoring its potential in mitigating stroke-induced brain injury. Our findings elucidate the critical role of PTBP1 in regulating circSCMH1 formation and demonstrate how this regulation contributes to astrocyte dysfunction. This insight highlights the significance of PTBP1 in the pathological mechanisms following ischemic stroke, offering new perspectives on novel therapeutic targets for stroke.

## Results

### CircSCMH1 downregulation and PTBP1 upregulation after stroke

**Figure 1:**
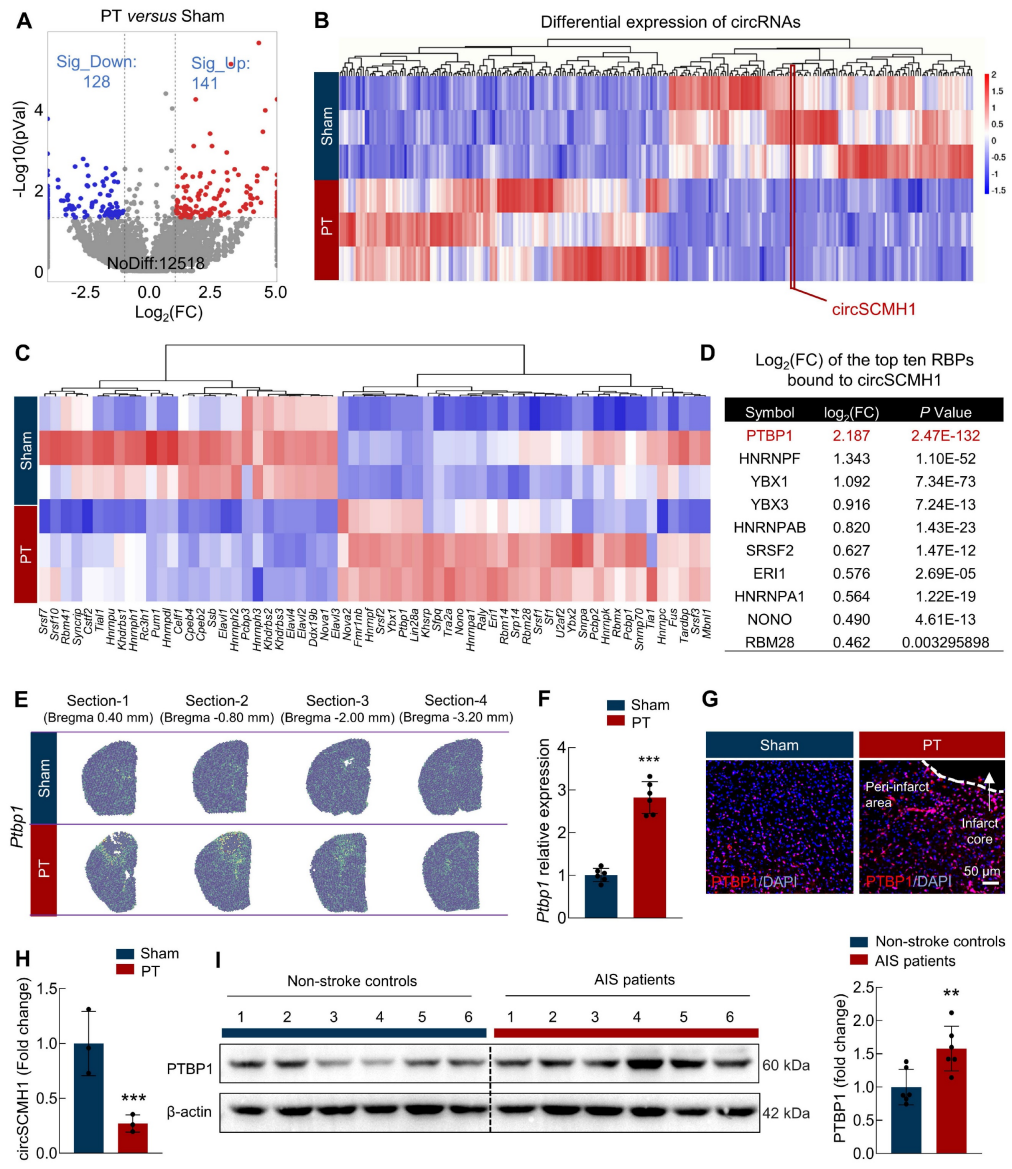
** PTBP1 functions as an RNA-binding protein for circSCMH1 and significantly increases in the infarct area after ischemic stroke. (A)** Significant upregulation and downregulation of circRNA were detected in the peri-infarct area of photothrombotic (PT) and sham mice at day 3 (fold change ≥ 1.5). n = 3 samples/group. **(B)** Distinct circRNA expression in the peri-infarct area of PT mice compared with that in sham mice (fold change ≥1.5, *P* < 0.05). n = 3 samples/group.** (C)** Microarray heat map representing distinct gene expression values in the peri-infarct area from the PT and sham mice at day 3 (adjusted *P <* 0.05). n = 3 samples/group. **(D)** The list of the top ten RBPs bound to circSCMH1. **(E)** Spatially resolved heatmaps from PT section-1, section-2, section-3, and section-4 showing the spatial expression patterns of *Ptbp1*.** (F)** The expression of *Ptbp1* in the peri-infarct area of PT mice compared with that in sham mice by real-time PCR. n = 6 samples/group. ^***^P < 0.001 versus Sham group. **(G)** The immunofluorescence staining of PTBP1 in the peri-infarct area of PT mice compared with that in sham mice. Scale bar = 50 μm. **(H)** The expression of circSCMH1 by real-time PCR at day 3. n = 3 samples/group. ^***^P < 0.001 versus Sham group. **(I)** The expression of PTBP1 in the brains of non-stroke controls and AIS patients was measured by western blotting. n = 6/group. All data are representative of at least 3 independent experiments. ^**^P < 0.01 versus AIS patients' group. Data are represented as means ± SD.

To identify key RBPs responsible for the downregulation of circSCMH1 following ischemic stroke, we first profiled circRNA and mRNA expression in the peri-infarct regions of photothrombotic (PT) stroke mice using RNA sequencing. Compared to the sham group, we found that 141 circRNAs were upregulated and 128 circRNAs were downregulated in the PT group** (Figure 1A)**. Critically, circSCMH1 was significantly downregulated in the PT group **(Figure 1B)**. We then employed the eCLIP database in conjunction with FIMO-based motif scanning to examine RBP binding sites within 1,000 nucleotides of the 5′ and 3′ flanking exon-intron boundaries of circularized exons. By integrating these findings with transcriptomic sequencing data, we identified 64 RBPs that could bind to the circSCMH1 precursor with significant differential expression: 41 RBPs were significantly upregulated, while 23 were significantly downregulated **(Figure 1C)**. PTBP1 emerged as the top RBP interacting with circSCMH1, with its levels significantly elevated in the PT group, according to transcriptome sequencing data **(Figure 1D)**. Spatial transcriptomics further revealed *Ptbp1* expression accumulation in the peri-infarct region of PT stroke mice **(Figure 1E)**. This expression pattern was further confirmed by real-time PCR and immunofluorescence, which showed increased level of *Ptbp1* mRNA and PTBP1 protein in the peri-infarct area after PT **(Figure 1F-G)** and transient middle cerebral artery occlusion (tMCAO) **(Figure S1A-B).** Furthermore, our examination of the peri-infarct brain tissue at day 3 post-stroke revealed a significant decrease in circSCMH1 levels, concurrent with a progressive increase in PTBP1 levels as the stroke evolved** (Figure 1H)**. We also collected somatosensory cortex tissue samples from acute ischemic stroke (AIS) patients through the Chinese Brain Bank Center (CBBC). Subsequent immunoblotting analysis revealed elevated PTBP1 levels in the AIS patient samples compared to those of non-stroke controls** (Figure 1I)**. Taken together, these findings demonstrate elevated PTBP1 levels and diminished circSCMH1 expression post-stroke, suggesting a potential regulatory mechanism involving circSCMH1 biogenesis in stroke pathology.

### PTBP1 exhibits significant increase specifically in astrocytes after stroke

**Figure 2:**
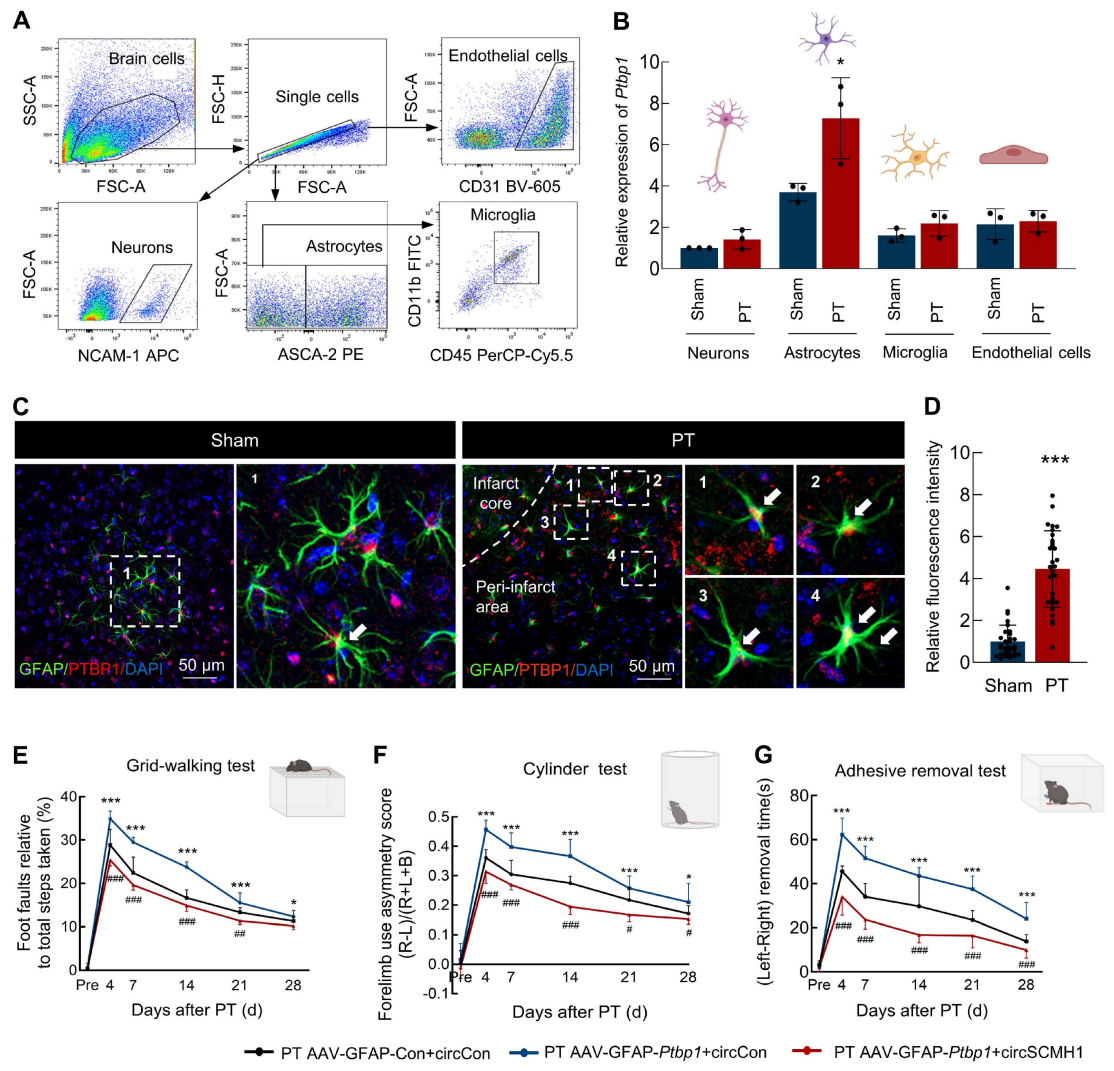
** PTBP1 exhibits a significant increase specifically in astrocytes in a PT stroke mouse model. (A)** Schematic of neurons, astrocytes, microglia, and endothelial cells isolation from PT and Sham groups.** (B)** Relative expression of *Ptbp1* in the sorted cells as determined by real-time PCR. n = 3 samples/group. ^*^P < 0.05 versus Sham group (Astrocytes). **(C-D)** The immunofluorescence staining of PTBP1 in astrocytes on the peri-infarct area of PT mice compared with that in sham mice, ^***^P < 0.001 versus Sham group. **(E-G)** circSCMH1 improved behavioral damage caused by AAV-GFAP-*Ptbp1* after stroke, as measured by the grid walking test **(E)**, cylinder test **(F)**, and adhesive removal test **(G)**. ^*^P < 0.05, ^***^P < 0.001 versus PT AAV-GFAP-Con+circCon group. ^#^*P <* 0.05, ^##^*P <* 0.01, ^###^*P <* 0.001 versus PT AAV-GFAP-*Ptbp1*+circCon. Data are represented as means ± SD. Con, Control.

Our previous research has demonstrated that following ischemic stroke, the expression of circSCMH1 is downregulated in various cell types, including neurons, astrocytes, vascular endothelial cells, and microglia^4^-^6^, ^14^. In order to examine the *Ptbp1* expression changes across major cell types in the ischemic brain, we conducted a detailed analysis using flow cytometry sorting to examine neurons, astrocytes, microglia, and endothelial cells **(Figure 2A)**. Our findings revealed that *Ptpb1* expressed in neurons, astrocytes, microglia, and endothelial cells, with the most substantial increase observed in astrocytes **(Figure 2B)**. Importantly, our results demonstrated a significant elevation in *Ptbp1* expression specifically within astrocytes following a stroke, while no significant changes in expression were observed in other cell types before or after the stroke** (Figure 2B)**. Immunofluorescence staining further confirmed that the PTBP1 is significantly increased in astrocytes following ischemic stroke, evidenced by the enhanced colocalization of PTBP1 with the astrocyte marker GFAP in the peri-infarct region in PT **(Figure 2C-D)** and tMCAO stroke model** (Figure S1C-D)**. The results collectively indicate that astrocytes are the primary cell type exhibiting significant upregulation of *Ptbp1* mRNA and PTBP1 protein after ischemic stroke, suggesting a potentially crucial role for astrocytic PTBP1 in regulating circSCMH1 expression in response to stroke-induced injury. Additionally, specific overexpression of *Ptbp1* in astrocytes exacerbated behavioral deficits in mice, whereas circSCMH1 restored motor function impairments induced by *Ptbp1* overexpression, as demonstrated by grid walking, cylinder, and adhesive removal tests **(Figure 2E-G)**. Overall, circSCMH1 counteracts the detrimental effects of *Ptbp1* overexpression on motor recovery in PT mice.

### PTBP1 promotes astrocyte activation by suppressing circSCMH1 expression

**Figure 3:**
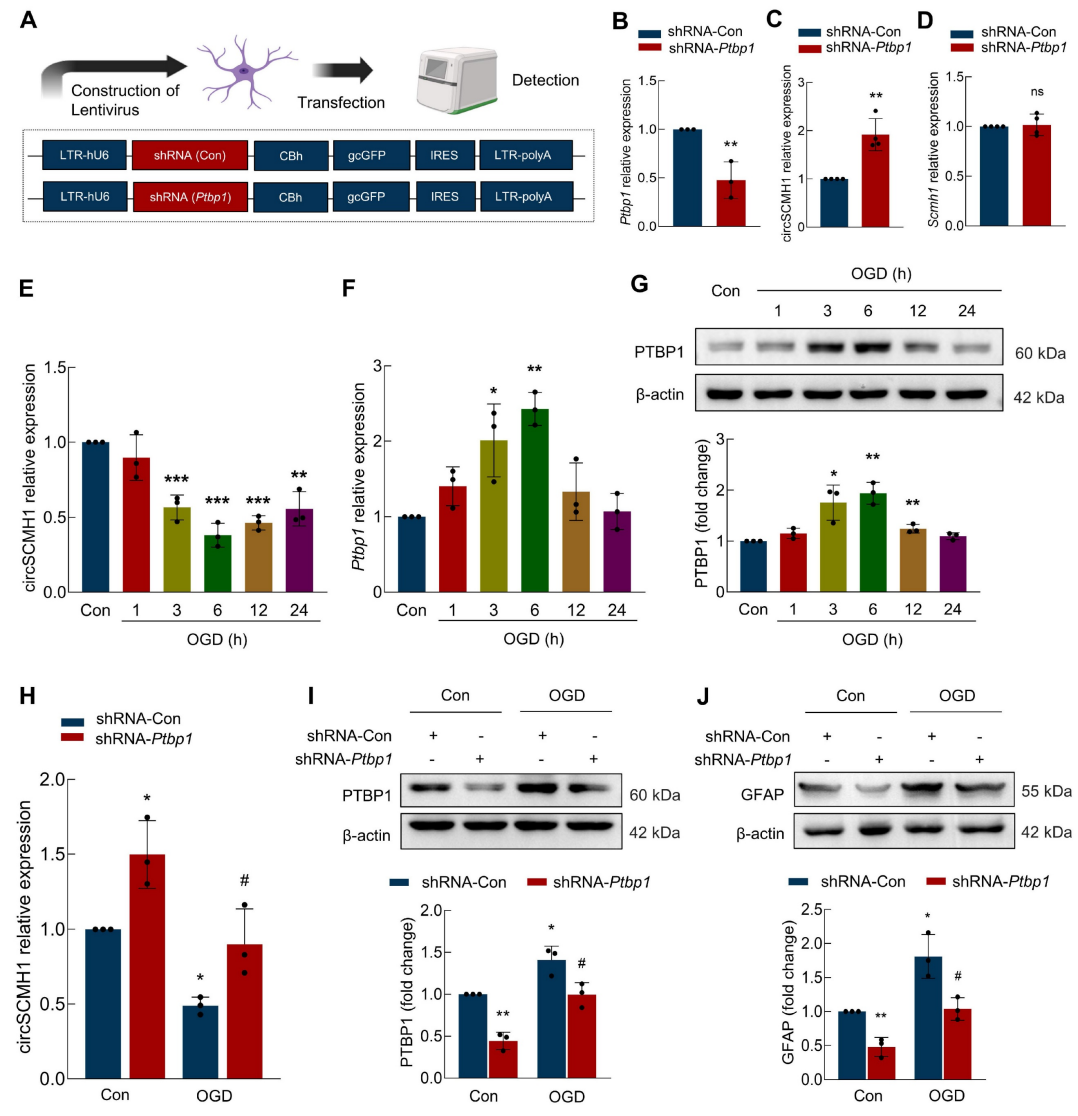
** PTBP1 influences astrocyte function by regulating circSCMH1. (A)** Schematic of lentivirus of shRNA-Con and shRNA-*Ptbp1*. **(B)** The level of *Ptbp1* was decreased in primary mouse astrocytes transduced with shRNA-*Ptbp1*. ^**^P < 0.01 versus shRNA-Con group.** (C)** The level of circSCMH1 was increased in primary mouse astrocytes transduced with shRNA-*Ptbp1*. ^**^P < 0.01 versus shRNA-Con group.** (D)** The level of *Scmh1* in primary mouse astrocytes transduced with shRNA-*Ptbp1*.** (E)** The expression of circSCMH1 under OGD/R at 0, 1, 3, 6, 12, and 24 h by real-time PCR. ^**^P < 0.01, ^***^P < 0.001 versus Con group.** (F)** The expression of *Ptbp1* under OGD/R at 0, 1, 3, 6, 12, and 24 h by real-time PCR. ^*^P < 0.05, ^**^P < 0.01 versus Con group.** (G)** Western blotting analysis of PTBP1 under OGD/R at 0, 1, 3, 6, 12, and 24 h. ^*^P < 0.05, ^**^P < 0.01 versus Con group. **(H)** The expression of circSCMH1 under OGD/R by real-time PCR in primary mouse astrocytes transduced with shRNA-*Ptbp1*. ^*^P < 0.05 versus Con+shRNA-Con group. ^#^*P <* 0.05 versus OGD+shRNA-Con group. **(I)** Western blotting analysis of PTBP1 under OGD/R in primary mouse astrocytes transduced with shRNA-*Ptbp1*. ^*^P < 0.05, ^**^P < 0.01 versus Con+shRNA-Con group. ^#^*P <* 0.05 versus OGD+shRNA-Con group.** (J)** Western blotting analysis of GFAP under OGD in primary mouse astrocytes transduced with shRNA-*Ptbp1*. ^*^P < 0.05, ^**^P < 0.01 versus Con+shRNA-Con group. ^#^*P <* 0.05 versus OGD+shRNA-Con group. All data are representative of at least 3 independent experiments. Data are represented as means ± SD. Con, Control. OGD, Oxygen and glucose deprivation. ns, no significant.

Observing upregulated circSCMH1 in astrocytes post-stroke, we thus investigated whether PTBP1 modulation alters astrocyte reactivity. Primary astrocytes were transfected with a lentivirus carrying shRNA targeting *Ptbp1* **(Figure 3A)**, which led to a significant reduction in *Ptbp1* expression **(Figure 3B)**. We then examined the impact of PTBP1 on circSCMH1 and *Scmh1* expression levels, found that shRNA-*Ptbp1* treatment resulted in a substantial increase in circSCMH1 levels, while *Scmh1* mRNA expression remained unchanged **(Figure 3C-D)**. These findings suggest that PTBP1 specifically regulates circSCMH1 without affecting the expression of its precursor gene. Further analysis revealed that exposing astrocytes to oxygen-glucose deprivation (OGD), a model for ischemic stroke, significantly reduced circSCMH1 levels, while *Ptbp1* levels increased over time, peaking at 6 hours post-treatment** (Figure 3E-F)**. Western blot analysis corroborated these changes, confirming PTBP1 upregulation following OGD treatment** (Figure 3G)**. Notably, introducing shRNA-*Ptbp1* significantly reversed the OGD-induced decrease in circSCMH1 levels** (Figure 3H)**, suggesting that *Ptbp1* inhibition can rescue circSCMH1 loss under ischemic conditions. Moreover, shRNA-*Ptbp1* treatment mitigated OGD-induced increases in PTBP1 and the astrocyte activation marker GFAP** (Figure 3I-J)**, indicating the crucial role of PTBP1 in astrocyte dysfunction following ischemia. These findings highlight the potential of targeting PTBP1 as a strategy to modulate circSCMH1 levels and reduce astrocyte activation during ischemic brain injury.

### PTBP1 binds to the Δ800-882 segment in the downstream intronic region of circSCMH1

**Figure 4:**
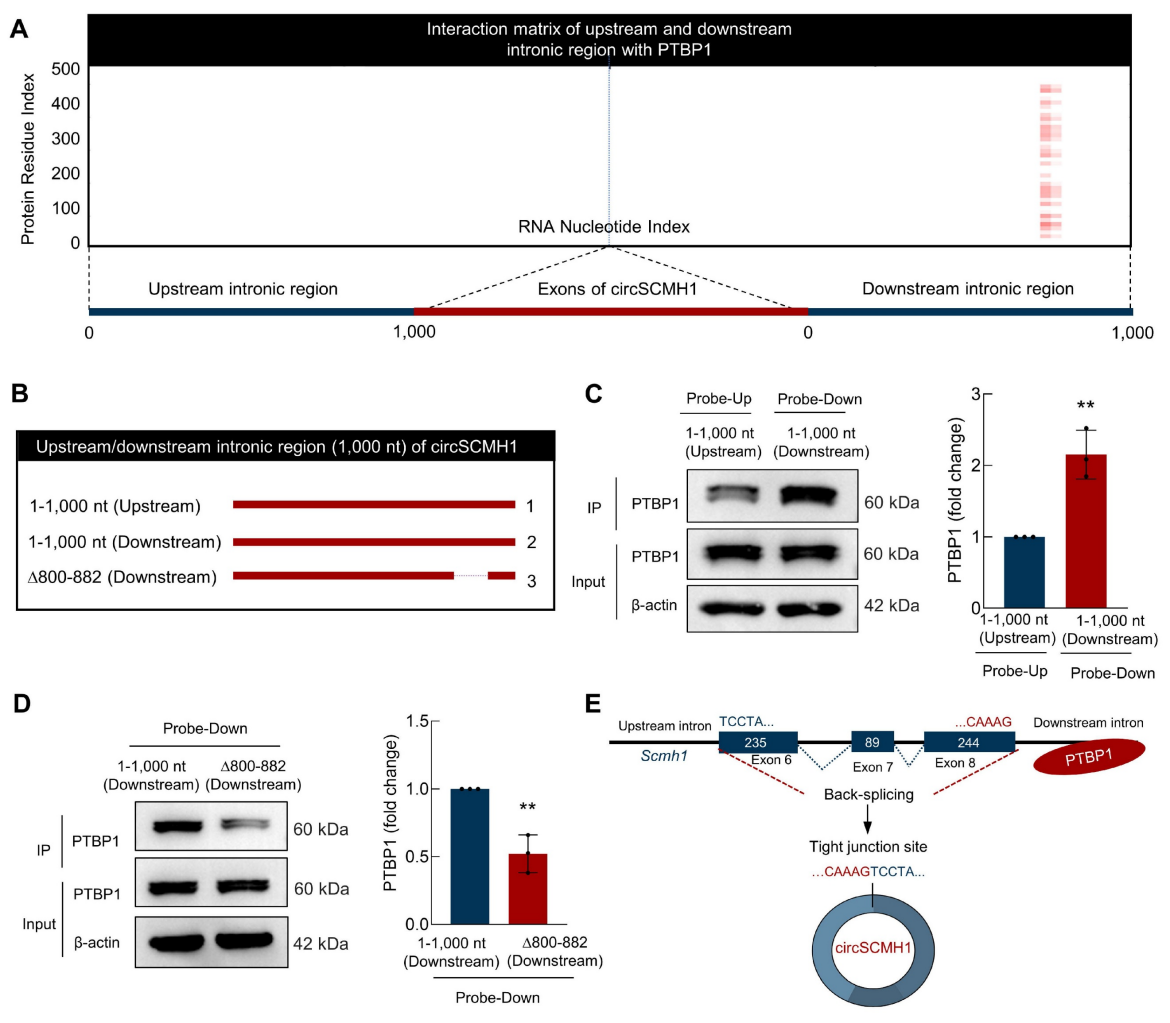
** PTBP1 binds to the downstream ∆800-882 region of circSCMH1 downstream intronic region. (A)** Interaction profiles of PTBP1 with circSCMH1 predicted by catRAPID. **(B-D)** Serial deletions of circSCMH1 were utilized in RNA pulldown assays to identify regions required for the circSCMH1 and PTBP1 interactions. ^**^P < 0.01 versus 1-1,000 nt (Upstream) Probe-Up group **(C)**. ^**^P < 0.01 versus 1-1,000 nt (Downstream) Probe-Down group **(D). (E)** Schematic of PTBP1 Inhibits the reverse splicing of circSCMH1. All data are representative of at least 3 independent experiments. Data are represented as means ± SD.

To identify the binding sites of PTBP1 in the flanking intronic sequences of circSCMH1, we employed the catRAPID algorithm^15^ to predict and analyze potential binding sites between PTBP1 and circSCMH1, revealed a higher probability of PTBP1 binding to the site of 800-882 in downstream intronic sequences **(Figure 4A)**. This computational approach provided a foundation for subsequent experimental validation and enabled us to assess the interaction propensities between these two molecules. Thus, we constructed a series of truncated circSCMH1 plasmids, including Upstream-1-1,000 nt, Downstream-1-1,000 nt, and Downstream-∆800-882, to investigate the specific regions responsible for PTBP1 binding **(Figure 4B)**. Firstly, RNA pulldown assays showed that the downstream intronic region binds more strongly to PTBP1 compared to the upstream intronic region **(Figure 4C)**. Furthermore, deletion assays targeting the 800-882 nt segment of circSCMH1's downstream intronic region identified this sequence as essential for PTBP1 binding, with the isolated fragment demonstrating sufficient binding capacity **(Figure 4D)**. In conclusion, through a combination of bioinformatics prediction and experimental validation, we successfully identified the 800-882 site in the downstream intronic sequence of circSCMH1 as the critical region for PTBP1 binding **(Figure 4D)**, laying the foundation for further investigation of the interaction mechanism between circSCMH1 and PTBP1 **(Figure 4E)**.

### The ∆RRM4 domain of PTBP1 binds to the Scmh1 precursor and inhibits the reverse splicing of circSCMH1

**Figure 5:**
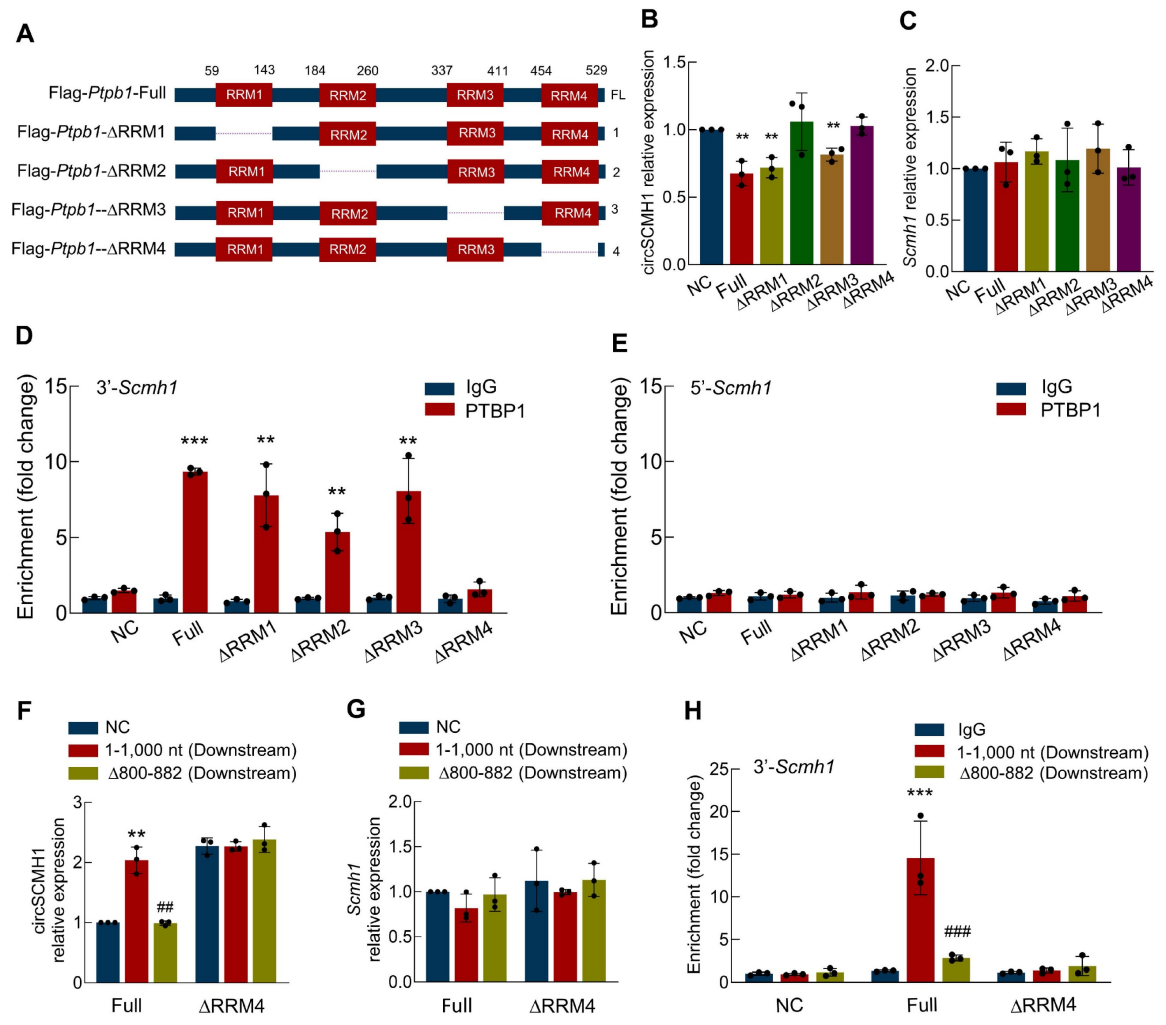
**The ∆RRM4 domain of PTBP1 binds to the *Scmh1* precursor and inhibits the reverse splicing of circSCMH1. (A)** Schematic of serial deletions of PTBP1 (Flag-PTBP1-Full, Flag-PTBP1-∆RRM3, Flag-PTBP1-∆RRM2, Flag-PTBP1-∆RRM3, Flag-PTBP1-∆RRM4).** (B-C)** The expression of circSCMH1**(B)** and *Scmh1* **(C)** in primary mouse astrocytes transduced with Flag-PTBP1-Full, Flag-PTBP1-∆RRM1, Flag-PTBP1-∆RRM2, Flag-PTBP1-∆RRM3, and Flag-PTBP1-∆RRM4 by real-time PCR. Data were presented as mean ± SD of 3 independent experiments. ^**^P < 0.01 versus NC group. **(D)** Interaction between 3'-*Scmh1* and PTBP1 validated by RNA immunoprecipitation (RIP) in primary mouse astrocytes. Data were presented as mean ± SD of 3 independent experiments. ^**^P < 0.01, ^***^P < 0.001 versus IgG group.** (E)** Interaction between 5'-*Scmh1* and PTBP1 validated by RNA immunoprecipitation (RIP) in primary mouse astrocytes. Data were presented as mean ± SD of 3 independent experiments. **(F-G)** The expression of circSCMH1 **(F)** and *Scmh1* **(G)** in primary mouse astrocytes transduced with Flag-PTBP1-Full and Flag-PTBP1-∆RRM4 by real-time PCR. ^**^P < 0.01 versus Full+NC group, ^##^*P* < 0.01 versus Full+1-1,000 nt group. **(H)** Interaction between 3'-*Scmh1* and PTBP1 validated by RIP in primary mouse astrocytes.^ ***^P < 0.001 versus Full+IgG group. ^###^*P* < 0.001 versus Full+1-1,000 nt (Downstream) group. Data were presented as mean ± SD of 3 independent experiments.

Having identified the ∆800-882 region of circSCMH1 as the interaction site with PTBP1, we sought to determine the specific regions of PTBP1 responsible for binding to circSCMH1. To this end, we constructed a series of truncated PTBP1 plasmids: Flag-PTBP1-Full, Flag-PTBP1-∆RRM1, Flag-PTBP1-∆RRM2, Flag-PTBP1-∆RRM3, and Flag-PTBP1-∆RRM4** (Figure 5A and Figure S2)**. These plasmids were transfected into astrocytes, and real-time PCR was subsequently employed to quantify the expression levels of circSCMH1. Our results demonstrated that PTBP1-∆RRM2 and PTBP1-∆RRM4 effectively prevented the decrease in circSCMH1 expression, while PTBP1-∆RRM1 and PTBP1-∆RRM3 showed no significant impact **(Figure 5B)**. Importantly, transfection with these plasmids did not alter the expression of linear *Scmh1* **(Figure 5C)**. Next, we employed RNA Immunoprecipitation (RIP) to measure the levels of the 3'-circSCMH1 and 5'-circSCMH1 flanking intronic regions following transfection with a series of PTBP1 truncated plasmids. The results indicated that PTBP1-∆RRM1, PTBP1-∆RRM2, and PTBP1-∆RRM3, but not PTBP1-∆RRM4, bind to the 3'-circSCMH1 flanking intronic region **(Figure 5D)**. This suggests that PTBP1-∆RRM4 is the crucial region required for binding to the precursor of circSCMH1. In contrast, no such effects were observed with the 5'-circSCMH1 flanking intronic region **(Figure 5E)**. The results presented above confirmed the core binding region between the circSCMH1 precursor and PTBP1, which led us to proceed with co-transfection experiments. Through real-time PCR analysis, we observed that downstream-∆800-882 markedly reduced the expression of circSCMH1 mediated by PTBP1 compared with downstream-1-1,000, while PTBP1-∆RRM4 did not affect the expression of circSCMH1 **(Figure 5F)**. In contrast, the expression of linear *Scmh1* remained unaffected **(Figure 5G)**. RIP assays further corroborated that both downstream-∆800-882 and PTBP1-∆RRM4 significantly attenuated the binding affinity of PTBP1 to circSCMH1 **(Figure 5H)**. Overall, these results demonstrate that the RRM4 domain of PTBP1 is crucial for binding to the ∆800-882 region of the circSCMH1 precursor, specifically interacting with the 3'-flanking intronic region. This interaction plays a key role in regulating circSCMH1 expression without affecting linear *Scmh1* levels, demonstrating the specificity and importance of this binding mechanism in circular RNA biogenesis.

### Downregulation of PTBP1 significantly inhibits the astrocyte activation and promotes the function recovery after ischemic stroke

**Figure 6:**
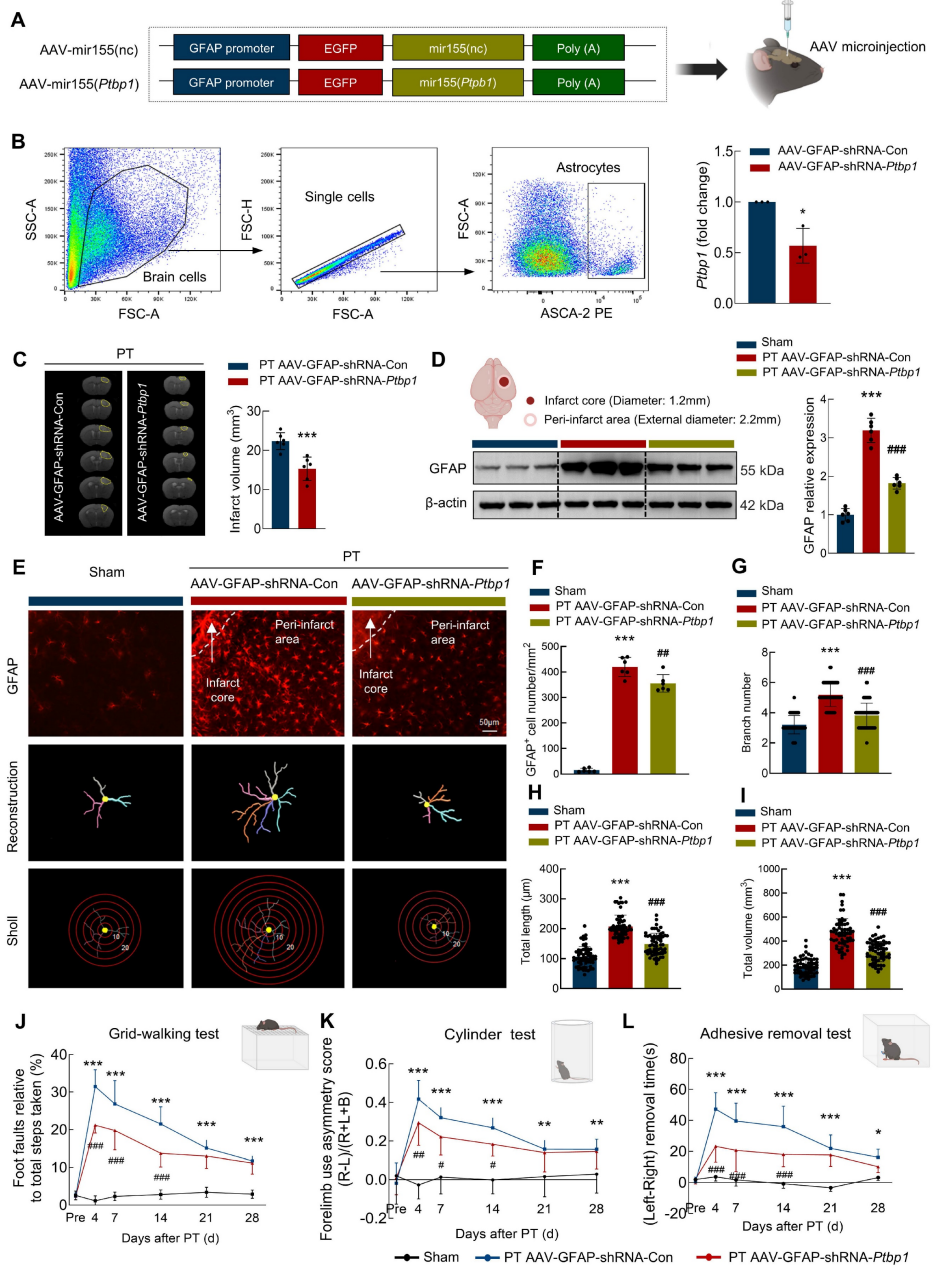
** Downregulating astrocytic PTBP1 significantly inhibits the astrocyte activation and promotes the function recovery after ischemic stroke. (A)** Schematic diagram of AAV-GFAP-shRNA-*Ptbp1* construction. **(B)** Astrocytes were sorted from brain tissues of C57BL/6 mice after AAV-GFAP-shRNA-*Ptbp1* injection. Relative expression of *Ptbp1* in the sorted cells was determined by qPCR. n = 3/group. ^*^P < 0.05 versus AAV-GFAP-shRNA-Con group.** (C)** Representative T2-weighted MRI images of AAV-GFAP-shRNA-Con and AAV-GFAP-shRNA-*Ptbp1* groups 1 days after PT, followed by an analysis of infarct volume. The dashed line denotes the infarct area. n = 6 mice/group. ^***^P < 0.001 versus PT+AAV-GFAP-shRNA-Con group. **(D)** Western blot analysis of GFAP expression. Three representative immunoblots from 6 mice/group are presented. ^***^P < 0.001 versus Sham group. ^###^*P* < 0.001 versus PT+AAV-GFAP-shRNA-Con group.** (E-I)** Effect of PTBP1 on the astrocyte activation after PT stroke model. Representative images of astrocyte immunostaining for GFAP in the peri-infarct area, followed by 3D reconstruction and Sholl analysis **(E)**, GFAP^+^ cell number **(F)**, branch number **(G)**, total branch length **(H)**, and total branch volume **(I)**. Scale bars: 50 μm. n = 6 mice/group, 60 cells/group. ^***^P < 0.001 versus Sham group. ^##^*P* < 0.01, ^###^*P* < 0.001 versus PT+AAV-GFAP-shRNA-Con group. **(J-L)** AAV-GFAP-shRNA-*Ptbp1* improved behavioral recovery at different time points after stroke, as measured by the grid walking test **(J)**, cylinder test **(K)**, and adhesive removal test **(L)**. n = 12/group. All data are presented as the mean ± SD. ^*^P < 0.05, ^**^P < 0.01, ^***^P < 0.001 versus Sham group, ^#^*P <* 0.05, ^##^*P <* 0.01, ^###^*P <* 0.001 versus PT+AAV-GFAP-shRNA-Con group.

To validate the function of PTBP1 in astrocytes after PT stroke mouse model, we microinjected AAV-GFAP-shRNA-Con or AAV-GFAP-shRNA-*Ptbp1* into the right lateral ventricle of mice **(Figure 6A)**. Three weeks post-injection, transduction analysis confirmed significantly reduced *Ptbp1* expression in AAV-GFAP-shRNA-*Ptbp1*-treated mice versus shRNA controls **(Figure 6B)**. Subsequent Magnetic Resonance Imaging (MRI) quantification demonstrated substantially decreased ischemic lesion volume following AAV-GFAP-shRNA-*Ptbp1* administration **(Figure 6C).** Moreover, this intervention attenuated stroke-induced GFAP upregulation in PT mice **(Figure 6D)**. Although specific knockdown of *Ptbp1* in astrocytes ameliorated behavioral deficits in mice, pharmacological inhibition of astrocytic function using rAAV-GfaABC1D-hM4D(Gi)-mCherry abolished this therapeutic effect **(Figure S3)**. Notably, loss of astrocytic function completely attenuated the behavioral improvement mediated by AAV-GFAP-shRNA-*Ptbp1*, suggesting intact astrocyte activity is essential for the *Ptbp1* knockdown-mediated therapeutic outcomes. To further investigate the impact of AAV-GFAP-shRNA-*Ptbp1* on astrocyte morphology, we performed immunostaining for GFAP and conducted 3D reconstruction **(Figure 6E)**. Sholl analysis revealed that PT stroke induced astrocyte activation. Importantly, AAV-GFAP-shRNA-*Ptbp1* treatment significantly alleviated these morphological deficits **(Figure 6F-6I)**. Collectively, these findings suggest that the abnormal upregulation of PTBP1 in astrocytes may play a critical role in the pathogenesis and progression of stroke.

Next, we investigated the impact of PTBP1 on the functional recovery of stroke mice. Pre-trained male mice underwent PT followed by microinjection. As illustrated in **Figure 6J**, animals treated with AAV-GFAP-shRNA-*Ptbp1* exhibited significantly improved performance in the grid-walking test, demonstrating reduced foot faults at days 4, 7, 14, 21, and 28 post-PT surgery compared to the AAV-GFAP-shRNA-Con group. Further evidence of a therapeutic effect was found in the cylinder test, which revealed significant differences between the two groups **(Figure 6K).** Consistent results were observed in the adhesive removal test, where mice injected with AAV-GFAP-shRNA-*Ptbp1* displayed reduced bias at the same time points following PT stroke** (Figure 6L)**. Consistent behavioral outcomes were observed in the tMCAO model **(Figure S1E-G).** Additionally, non-cell-specific *Ptbp1* knockdown approaches demonstrated comparable efficacy **(Figure S4-5)**. Collectively, these results showed that targeting PTBP1 in astrocytes significantly enhances functional recovery after stroke by mitigating astrocyte activation and structural deficits.

## Discussion

This study systematically characterizes a previously unrecognized PTBP1-circSCMH1 regulatory axis that critically modulates post-ischemic neuronal survival and functional recovery. Following an ischemic stroke, the expression of PTBP1 is significantly upregulated, particularly in astrocytes. Mechanistic investigations demonstrate that PTBP1 interacts with the circSCMH1 precursor, specifically binding to the 800-882 region in the downstream intronic sequence via its RRM4 domain, thereby inhibiting circSCMH1 biogenesis. Utilizing both *in vivo* and *in vitro* models, the study provides compelling evidence that downregulating PTBP1 not only restores circSCMH1 expression but also reduces astrocyte activation, leading to improved functional outcomes. The innovative aspect of this study lies in its identification of PTBP1 as a crucial modulator of circSCMH1 biogenesis and astrocyte activation. By unveiling this intricate regulatory pathway, our study offers a potential therapeutic target for enhancing recovery after ischemic brain injury.

As a focal point of our research, the circular RNA molecule circSCMH1 has shown significant promise. Its efficacy has been thoroughly validated in non-human primate and mouse studies^4^. And the cellular and molecular mechanisms by which it facilitates brain repair after ischemic stroke have been extensively detailed, including the enhancement of neuronal plasticity, promotion of vascular repair, and an increase in astrocytic microdomain calcium activities^4^-^6^. As we continue to advance the development of nucleic acid therapeutics based on circSCMH1, it is crucial to elucidate the underlying mechanism responsible for the reduced expression of circSCMH1 following ischemic stroke. To address this fundamental scientific question, we employed a comprehensive approach integrating multiple analytical methods. We combined data from the eCLIP database with FIMO-based motif scanning to analyze RBP binding sites within the flanking intronic regions of circSCMH1. By integrating these findings with transcriptomic sequencing data, we identified PTBP1 as a potential key regulator of circSCMH1 biogenesis.

PTBP1 has garnered considerable interest in recent years owing to its pivotal role in RNA splicing and gene expression regulation^16^-^18^. Altered PTBP1 expression affects the splicing of numerous RNAs, reported to play an important role in tumors^16^, neurogenesis^19^, and glioblastoma progression^20^. Beyond its role in mRNA splicing, PTBP1 is also a critical factor in the biogenesis and regulation of circRNAs, a class of non-coding RNAs that have emerged as important regulators of gene expression^21^. Additionally, the expression of *Ptbp1* is positively correlated with the progression and poor prognosis of various diseases, indicating that elevated *Ptbp1* has a detrimental impact on physiological and pathological processes^22^. However, its involvement in ischemic stroke remains unexplored^23^. Ischemic stroke induced substantial PTBP1 upregulation in peri-infarct zones, as demonstrated in our study. This increase in PTBP1 inhibits the back-splicing process involved in circSCMH1 biogenesis. We found that PTBP1 specifically binds to the downstream region of circSCMH1, particularly within the 800-882 nucleotide range, rather than to the upstream region or other downstream segments. Notably, the RRM2 and RRM4 of PTBP1 are critical for this binding interaction. Consequently, this binding leads to a reduction in circSCMH1 expression, which ultimately impacts downstream biological processes.

Our bioinformatic analysis predicted interactions between circSCMH1 and multiple RBPs (HNRNPF, YBX1, and YBX3), implying potential alternative pathways in circSCMH1 biogenesis regulation. It is plausible that these proteins, either independently or through interactions with PTBP1, could influence stroke prognosis. This potential connection will be the focus of our future research. Additionally, our study demonstrated that PTBP1 is highly expressed in astrocytes following ischemic stroke, elucidating the specific molecular mechanism responsible for the reduction of circSCMH1 in astrocytes. However, the role of circSCMH1 in the onset and progression of stroke extends beyond glial cells, playing a significant part in neurons and endothelial cells as well^4^, ^5^. Notably, our previous studies have experimentally validated multiple downstream mechanisms through which circSCMH1 exerts neuroprotective effects: (1) enhancing neuronal plasticity while suppressing glial activation and peripheral immune cell infiltration by binding to the transcription factor MeCP2 and relieving its repressive effects on target genes 4; (2) promoting vascular repair via FTO-mediated m^6^A demethylation of *Plpp3* mRNA in endothelial cells^5^; (3) facilitating behavioral recovery through formation of DDX1-dependent membrane-associated RNA vesicles (MARVs) that regulate astrocytic microdomain Ca^2+^ transients 6; and (4) when delivered via RVG-engineered extracellular vesicles, enhancing mitochondrial fusion while inhibiting mitophagy through STAT5B-mediated suppression of KMO expression 14.Therefore, further investigation is warranted to determine whether circSCMH1 biosynthesis involves cell type-specific regulatory mechanisms or potential cross-cellular interactions that collectively contribute to its global downregulation post-stroke - a critical question for comprehensively understanding the regulatory networks underlying brain repair.

This research unveils a novel regulatory mechanism in ischemic stroke, highlighting how PTBP1 upregulation, especially in astrocytes, inhibits the biogenesis of circSCMH1 by binding to its precursor. The study identifies the specific binding domains and demonstrates that downregulating PTBP1 restores circSCMH1 levels, reduces astrocyte activation, and enhances functional recovery after ischemic stroke. This work not only advances our understanding of circular RNA biogenesis in pathological conditions but also positions PTBP1 as a potential therapeutic target for improving stroke recovery. By bridging a critical gap in stroke research, it offers new insights into the molecular mechanisms of brain repair and opens avenues for innovative nucleic acid-based therapies in neurological disorders.

## Materials and Methods

### Animals

We randomly assigned male C57BL/6J mice (6-8 weeks old; GemPharmatech, Nanjing, China) to experimental groups.

### Ethics statement

All experiments involving animals were conducted according to the ethical policies and procedures approved by the Institutional Animal Care and Use Committee (IACUC) of the Southeast University (approval ID 20190222003) and performed in accordance with the Animal Research: Reporting of In Vivo Experiments (ARRIVE) guidelines.

### Photothrombosis (PT) stroke model

Photothrombosis of cortical microvessels was used to induce focal cortical ischemia in mice, as previously described^24^, ^25^. Mice were sedated using 2% isoflurane and secured in a stereotaxic instrument. During surgery, the rectal temperature of the mice was maintained at 37 ± 0.5°C using a homeothermic blanket. The skull was exposed through a midline skin incision, with connective tissues cleared and the surface kept dry. A cold light source attached to an opaque template with a 2-mm diameter, 12,000 lux illumination opening was positioned 1.5 mm lateral from the bregma. Rose Bengal solution (30 mg/kg, 330000-1G, Sigma) was administered by tail vein injection. The brain was illuminated for 5 min. This light stimulation resulted in the production of singlet oxygen from Rose Bengal, causing damage and obstruction of the vascular endothelium and ultimately leading to a focal cortical ischemic stroke.

### RT-PCR

According to the previously reported^26^, ^27^, RNA was extracted using TRIzol reagent (15596026, Invitrogen, USA) and reverse-transcribed with a HiScript Q RT SuperMix for qPCR Kit (R123-01, Vazyme, China). Subsequently, the RNA was quantified using SYBR Green Real-time PCR Master Mix (Q141-02, Vazyme, China). The cycle threshold was measured with the StepOneTM Real-Time PCR instrument (Applied Biosystems, Foster City, USA). **Table 1** presented the primer sequences.

### The AAV-GFAP-shRNA-*ptbp1* microinjection

The mice were microinjected with the AAV-GFAP-shRNA-*Ptbp1* virus or an AAV-GFAP-shRNA-Con (Shanghai GeneChem Co., Ltd., China) at the following coordinates: 0.3 mm posterior to the bregma and 1.0 mm lateral to the sagittal midline, with the injection depth being 2.2 mm from the skull surface (lateral ventricles).

### Behavioral tests

*The grid-walking task:* An elevated grid area of 32 cm-length x 20 cm-width made of 12 mm square wire mesh was used^25^. Each mouse was placed individually on the wire grid and allowed to freely move until a minimum of 100 steps have been taken by the left forelimb. A camera was positioned beneath the grid to record stepping errors (foot faults). The ratio was determined using the following formula: number of foot faults / (number of foot faults + number of non-faults) x100. *The cylinder test:* It encourages the use of forelimbs for vertical wall exploration/press in a cylinder^28^. Animals were placed inside a plastic cylinder (15 cm tall with a diameter of 10 cm) and videotaped for 5 min. The ratio was determined using the following formula: (number of right hand- number of left hand) / (number of right hand + number of left hand + number of both hand). *The adhesive removal somatosensory test:* As previously described 29, 30, two small adhesive paper dots (each 25 mm² in size) were applied as bilateral tactile stimuli to the distal-radial region of the wrist on each forelimb. The time it took for the mice to remove each stimulus was recorded over three trials per day, with individual trials spaced at least 5 min apart. The ratio was determined using the following formula: time of left hand- time of right hand.

### Flow cytometry

Cells were isolated from mice brain. The tissue was digested with 2 mg/ml papain (LS003119, Worthington, USA) at 37°C for 1 h. After filtering through the nylon mesh (70 μm), the cells were resuspended in a 30% Percoll density gradient (17-0891-09, GE Healthcare, USA) and centrifuged at 500 g for 25 min. The cells at the bottom were collected and washed in PBS with 2% FBS. They were subsequently blocked with FcR Blocking Reagent (130-092-575, Miltenyi Biotec, Germany). All cells were marked as previously described^28^, ^31^-^33^. The RNA extraction used RNeasy®-Micro Kit (74004, QIAGEN, Germany).

### Western blot analysis

All tissue samples were collected at 3 days post-stroke under deep anesthesia following perfusion with 0.1M PBS (pH 7.4), with proteins extracted using RIPA lysis buffer (Beyotime, Shanghai, China). Samples separated on sodium dodecyl sulfate-polyacrylamide gel electrophoresis (SDS-PAGE). Subsequently, proteins were transferred onto polyvinylidene fluoride (PVDF) membranes^34^. The PVDF membranes were blocked with 5%nonfat milk and probed with GFAP (60190-1-Ig, Proteintech, China), PTBP1 (12582-1-AP, Proteintech, China) and β-actin (66009-1-Ig, Proteintech, China) overnight at 4°C. Next, the membranes were incubated with HRP-conjugated Affinipure Goat Anti-Mouse IgG and Anti-Rabbit IgG (SA00001-1, SA00001-2, Proteintech, China). The results were then visualized using the Tanon 5200 system.

### Immunofluorescence staining test

In our previously studies^35^, the sections (30 μm) were incubated with H_2_O_2_ for 10 min, incubated with 0.3% Triton X-100 in PBS for 15 min, and then blocked with 10% normal goat serum (NGS) in 0.3% Triton X-100 for 1 h at 25°C. Then, the sections were incubated with the GFAP (G3893, Sigma Aldrich, USA) overnight. Next, the sections were washed, and incubated with Alexa Fluor 594 goat anti-rabbit IgG (A-11037, Thermo Fisher Scientific, USA) at 25 °C for 1 h.

### Oxygen and glucose deprivation (OGD) treatment

OGD was performed as previously described^36^. Briefly, cells were cultured with deoxygenated DMEM without glucose (Gibco, 11966-025) in an incubator (Waltham, USA) with premixed 5% CO_2_ and 95% N_2_ for 6 h. Control group cultures were cultured with normal neurobasal medium for the same incubation time.

### RNA immunoprecipitation

The samples were harvested and collected in ice-cold PBS. This suspension was then centrifuged at 1500 rpm for 5 min at 4°C. The resulting cell pellet was resuspended in an equal volume of complete RIP lysis buffer. RNA immunoprecipitation was carried out using the Magna RIPTM RNA-binding protein immunoprecipitation kit (17-700), the EZ-Magna RIPTM kit (17-701), or the Magna RIP quad kit (17-704), all provided by Millipore, USA.

### RNA pull-down assays

As previously reported^37^, cells were washed in ice-cold phosphate-buffered saline, lysed in 500 μl co-IP buffer (P0013F, Beyotime, China), and incubated with 6 μg biotin-labeled circSCMH1 probe against endogenous or ectopically expressed circSCMH1 at room temperature for 2 h. A total of 50 μl washed Streptavidin C1 magnetic beads (65002, Invitrogen, USA) were added to each binding reaction and further incubated at room temperature for another hour. The beads were washed 5 times with co-IP buffer to remove unbound proteins. Subsequently, the bound proteins from the pull-down materials were isolated and detected by western blot analysis.

### Statistics

All data are shown as mean ± SD. Statistical analysis was performed using GraphPad Prism 9.0. For comparisons between two groups, significance was determined using a two-tailed Student's t-test. For comparisons involving three or more groups, one- or two-way ANOVA followed by Holm-Sidak post hoc tests was utilized.

## Supplementary Material

Supplementary figures.

## Figures and Tables

**Table 1:** Information of PCR primers

List of oligonucleotide sequences	5' - 3'
circSCMH1-Forward	CTACTGGTGCCGCTTTGACT
circSCMH1-Reverse	GGCACCTGTCAATCCAACGA
Ptbp1-Forward	CAGCAGCCAATGGAAACGATA
Ptbp1-Reverse	GCAGCTTTCTGACATGGATGAC
Scmh1-Forward	ATGGTAGCTGGGATGTTAGGG
Scmh1-Reverse	TGAGCTTTCTGATGTTTCTGCTT
β-actin-Forward	GGCTGTATTCCCCTCCATCG
β-actin-Reverse	CCAGTTGGTAACAATGCCATGT
3'-Scmh1-Forward	ATGAGGGCTGACTCCTTAGGT
3'-Scmh1- Reverse	GGAGGCTGAGAATGACACCTT
5'-Scmh1-Forward	GACATCACCGGACACTCCTAC
5'-Scmh1- Reverse	AACCAGCCTTGCTTATTCCCT

**Table 2:** Plasmid construction

Name	Sequence	Length
pCMV3-SV40-mCherry(2A) Puro-Upstream (1-1000nt)	atgtacattgccacatgtttataagaaccaaaagctagagctaaactaaatggtctcttactcagcagctaaaggagatgtatatcagatccagggtcattacattttagaaatcacaagtgtttaaaattccagattttggaatattttcttacaaagtatctatggatttaacccatctaagcatgcaatttattattgtttcttacatgtcttatgcctctagtctcaaggtaattattatacaatattttaatagtctgtattttgacttcaacacgtcatgtaatgccaagtacagaattttcttgtaccagcatgtcagtgacaaagtttctcagtttgaatctcataatttcagattaggtatactttacttatattgatatatttagcaatatggagaatagttgtactaactaaaaaaattaataataaaaacagtactcctccatgattccatgtaagattagaaggggaggggctagcaaaggaaagtataatgaagcatgtgagaacttgaagaataatggtgtgttcattattttgattataatggtactttcagaggtaaatacagtacatgtaatgccatgtcaagacttagcaaagagtacagtttactgtatgtcaattatgttagttgattgaatattaacataaaaaattaacctctaatggtctgtgtggtagaattagaaaaggtggatgctcagcagacatcaccggacactcctaccatttcttatactggtaatgagtagatacttgatacgtcacaggaaagtaacttttttccttcattgtattttctcatgcttcatttgtgtaaaacctttcttaaatgttagggaataagcaaggctggttgtccctgccccaagtgtatagccagtttgaatccaaatgcttgtttttttcaagttctacctccctagcattcctattgacatgcagtttatccgtagtctgcctaattcccttgccttgttctcctcag	1000 nt
pCMV3-SV40-mCherry(2A) Puro-Downstream (1-1000nt)	Gtaaagagccacctgtagttgcctgaaagcagaccttagctaatacgaggaatcgtccaggtttagtgtgctcagttgtctttaccagcatgcttcactgtaggcaggagaggaatgagggctgactccttaggtacacaccaagggcttccagagaataatcactaacagatcaagagtaagatcaaattaagccagatatgaaatctggggaaaagtaaaaaattctctcttctaaatatcccaacctattctcaggcaattcctgcttttgcccctaacacatttaaaagtttgaaggtgtcattctcagcctccaagggttgagaattgtgtagtgagctgggaaacactgttgatgaagtagaacctttgggataaaaggacaggctctacctctgcctgccactagcagctaattcacctctaaacctcaaatgcctgtttataaaagaacacctacttcaaaaaattcctgagagaaataaatgaaatcacatggacaaaatgcccacatctgcaaaatagcaagctccaagacaatttctgttttatttctgtggctatttttttttcttttttgttttgtttgtttgttttcactttctttttttattagatattttcttcatttacatttcaaatgctacccccttttcctaatttcctcttcgaaaattccctatgccctcccactccccctgctccccaacccacccactccccgattcctggccctggcatttccctaaactggggcatataatcttcacaagacctagggcctctcctcccattgatggctgactaggccatcctctgctacataggcagctagagacacgagctctggaggcaggggcgggtactggttagttcatattgttgtttctcctatagggttgcagaccccttcagctccttgggtactttctccagctcctccattgggaccgtgtgttccatccaatagttgactgtgagcatccacttctgtatt	1000 nt
pCMV3-SV40-mCherry(2A) Puro-Downstream (∆800-882)	gtaaagagccacctgtagttgcctgaaagcagaccttagctaatacgaggaatcgtccaggtttagtgtgctcagttgtctttaccagcatgcttcactgtaggcaggagaggaatgagggctgactccttaggtacacaccaagggcttccagagaataatcactaacagatcaagagtaagatcaaattaagccagatatgaaatctggggaaaagtaaaaaattctctcttctaaatatcccaacctattctcaggcaattcctgcttttgcccctaacacatttaaaagtttgaaggtgtcattctcagcctccaagggttgagaattgtgtagtgagctgggaaacactgttgatgaagtagaacctttgggataaaaggacaggctctacctctgcctgccactagcagctaattcacctctaaacctcaaatgcctgtttataaaagaacacctacttcaaaaaattcctgagagaaataaatgaaatcacatggacaaaatgcccacatctgcaaaatagcaagctccaagacaatttctgttttatttctgtggctatttttttttcttttttgttttgtttgtttgttttcactttctttttttattagatattttcttcatttacatttcaaatgctacccccttttcctaatttcctcttcgaaaattccctatgccctcccactccccctgctccccaacccacccactccccgattcctggccctggcatttccctaaactggggcatataatcttcacaagacctagggcctctcctcccattgttgttgtttctcctatagggttgcagaccccttcagctccttgggtactttctccagctcctccattgggaccgtgtgttccatccaatagttgactgtgagcatccacttctgtatt	917 nt
pCMV3-SV40-EGFP(2A) Puro-c3×Flag-*Ptbp1*	GGTACCGCCACCatggacggcatcgtcccagacatagcagtcggtacaaagcggggatccgacgagctcttctccacgtgtgtcagcaacggccccttcatcatgagcagctctgcctcagcagccaatggaaacgatagcaagaagttcaaaggtgacaacaggagcgcaggagtcccttccagagtcatccatgtcagaaagctgcccagcgatgtcactgagggcgaggtcatctccctagggctgccctttggaaaggttaccaaccttctcatgctgaaggggaagaaccaggccttcattgagatgaacacagaggaggctgccaacactatggttaactactatacatcggtggcgccagtgcttcgtggacagcccatctacatccagttctccaaccacaaagagctcaagaccgacagctcgcccaaccaggcacgtgcccaggcagccctgcaggctgtaaactccgtccagtctggaaacctggccttggcagcgtccgctgctgccgtggatgcaggaatggcaatggcagggcagagcccagtgctcaggatcattgtggaaaaccttttctacccagtgaccctggacgtgctgcaccagatcttctctaagtttggcaccgtcctgaagatcatcacgttcaccaagaacaaccagttccaggcgctgctgcagtatgctgaccctgtgagcgcccagcatgccaagctgtccctggatggccagaacatctacaacgcctgctgcacgctgcgcatcgacttctccaagctcaccagtctcaatgtcaagtacaacaatgataagagcagagactacactcgacctgacctgccctctggagacagccagccttcactagaccagaccatggcagcagcctttggtgcgcccggcataatgtcagcctctccgtatgcaggagccgggttccctcccacctttgccatccctcaggccgcaggcctctctgtccctaatgtccatggagccttggcccccctggccatcccgtctgctgctgctgctgctgcggccagccgcattgccatcccagggttggcaggtgctgggaattctgtccttttggtcagcaatctgaaccctgagagagtcacaccccaaagcctctttattctcttcggcgtctacggtgatgtgcagcgggtgaagatcctgttcaataagaaggagaacgcacttgtgcagatggcagacggcagccaggcccagctggccatgagccacctgaacgggcacaagctgcacgggaagtcagtgcgcattacactgtccaagcatcagagtgtgcagctgcctcgggagggtcaggaggaccagggcctgaccaaggactatggcagctccccgctgcaccgcttcaagaaaccaggctccaagaacttccagaacatctttccaccctcagctaccctgcacctctccaacatcccgccctctgtgtcagaggacgacctcaagagcctcttctccagcaacggtggtgtggtcaaaggcttcaagttcttccagaaggaccgcaagatggcactgatccagatgggctctgtggaggaggctgtgcaggcgctgattgaactgcacaaccatgacctgggcgagaaccaccacctgcgagtgtccttttccaagtccaccatctaTCTAGA	1685 nt
pCMV3-SV40-EGFP(2A)Puro-c3×Flag-△*Ptbp1*(58-142)	GGTACCGCCACCatggacggcatcgtcccagacatagcagtcggtacaaagcggggatccgacgagctcttctccacgtgtgtcagcaacggccccttcatcatgagcagctctgcctcagcagccaatggaaacgatagcaagaagttcaaaggtgacaacaggagcgcaggagtcccttcccaggcacgtgcccaggcagccctgcaggctgtaaactccgtccagtctggaaacctggccttggcagcgtccgctgctgccgtggatgcaggaatggcaatggcagggcagagcccagtgctcaggatcattgtggaaaaccttttctacccagtgaccctggacgtgctgcaccagatcttctctaagtttggcaccgtcctgaagatcatcacgttcaccaagaacaaccagttccaggcgctgctgcagtatgctgaccctgtgagcgcccagcatgccaagctgtccctggatggccagaacatctacaacgcctgctgcacgctgcgcatcgacttctccaagctcaccagtctcaatgtcaagtacaacaatgataagagcagagactacactcgacctgacctgccctctggagacagccagccttcactagaccagaccatggcagcagcctttggtgcgcccggcataatgtcagcctctccgtatgcaggagccgggttccctcccacctttgccatccctcaggccgcaggcctctctgtccctaatgtccatggagccttggcccccctggccatcccgtctgctgctgctgctgctgcggccagccgcattgccatcccagggttggcaggtgctgggaattctgtccttttggtcagcaatctgaaccctgagagagtcacaccccaaagcctctttattctcttcggcgtctacggtgatgtgcagcgggtgaagatcctgttcaataagaaggagaacgcacttgtgcagatggcagacggcagccaggcccagctggccatgagccacctgaacgggcacaagctgcacgggaagtcagtgcgcattacactgtccaagcatcagagtgtgcagctgcctcgggagggtcaggaggaccagggcctgaccaaggactatggcagctccccgctgcaccgcttcaagaaaccaggctccaagaacttccagaacatctttccaccctcagctaccctgcacctctccaacatcccgccctctgtgtcagaggacgacctcaagagcctcttctccagcaacggtggtgtggtcaaaggcttcaagttcttccagaaggaccgcaagatggcactgatccagatgggctctgtggaggaggctgtgcaggcgctgattgaactgcacaaccatgacctgggcgagaaccaccacctgcgagtgtccttttccaagtccaccatctaTCTAGA	1430 nt
pCMV3-SV40-EGFP(2A) Puro-c3×Flag-△*Ptbp1*(183-259)	GGTACCGCCACCatggacggcatcgtcccagacatagcagtcggtacaaagcggggatccgacgagctcttctccacgtgtgtcagcaacggccccttcatcatgagcagctctgcctcagcagccaatggaaacgatagcaagaagttcaaaggtgacaacaggagcgcaggagtcccttccagagtcatccatgtcagaaagctgcccagcgatgtcactgagggcgaggtcatctccctagggctgccctttggaaaggttaccaaccttctcatgctgaaggggaagaaccaggccttcattgagatgaacacagaggaggctgccaacactatggttaactactatacatcggtggcgccagtgcttcgtggacagcccatctacatccagttctccaaccacaaagagctcaagaccgacagctcgcccaaccaggcacgtgcccaggcagccctgcaggctgtaaactccgtccagtctggaaacctggccttggcagcgtccgctgctgccgtggatgcaggaatggcaatggcagggcagagcccagtgaccagtctcaatgtcaagtacaacaatgataagagcagagactacactcgacctgacctgccctctggagacagccagccttcactagaccagaccatggcagcagcctttggtgcgcccggcataatgtcagcctctccgtatgcaggagccgggttccctcccacctttgccatccctcaggccgcaggcctctctgtccctaatgtccatggagccttggcccccctggccatcccgtctgctgctgctgctgctgcggccagccgcattgccatcccagggttggcaggtgctgggaattctgtccttttggtcagcaatctgaaccctgagagagtcacaccccaaagcctctttattctcttcggcgtctacggtgatgtgcagcgggtgaagatcctgttcaataagaaggagaacgcacttgtgcagatggcagacggcagccaggcccagctggccatgagccacctgaacgggcacaagctgcacgggaagtcagtgcgcattacactgtccaagcatcagagtgtgcagctgcctcgggagggtcaggaggaccagggcctgaccaaggactatggcagctccccgctgcaccgcttcaagaaaccaggctccaagaacttccagaacatctttccaccctcagctaccctgcacctctccaacatcccgccctctgtgtcagaggacgacctcaagagcctcttctccagcaacggtggtgtggtcaaaggcttcaagttcttccagaaggaccgcaagatggcactgatccagatgggctctgtggaggaggctgtgcaggcgctgattgaactgcacaaccatgacctgggcgagaaccaccacctgcgagtgtccttttccaagtccaccatctaTCTAGA	1454 nt
pCMV3-SV40-EGFP(2A) Puro-c3×Flag-△*Ptbp1*(361-412)	GGTACCGCCACCatggacggcatcgtcccagacatagcagtcggtacaaagcggggatccgacgagctcttctccacgtgtgtcagcaacggccccttcatcatgagcagctctgcctcagcagccaatggaaacgatagcaagaagttcaaaggtgacaacaggagcgcaggagtcccttccagagtcatccatgtcagaaagctgcccagcgatgtcactgagggcgaggtcatctccctagggctgccctttggaaaggttaccaaccttctcatgctgaaggggaagaaccaggccttcattgagatgaacacagaggaggctgccaacactatggttaactactatacatcggtggcgccagtgcttcgtggacagcccatctacatccagttctccaaccacaaagagctcaagaccgacagctcgcccaaccaggcacgtgcccaggcagccctgcaggctgtaaactccgtccagtctggaaacctggccttggcagcgtccgctgctgccgtggatgcaggaatggcaatggcagggcagagcccagtgctcaggatcattgtggaaaaccttttctacccagtgaccctggacgtgctgcaccagatcttctctaagtttggcaccgtcctgaagatcatcacgttcaccaagaacaaccagttccaggcgctgctgcagtatgctgaccctgtgagcgcccagcatgccaagctgtccctggatggccagaacatctacaacgcctgctgcacgctgcgcatcgacttctccaagctcaccagtctcaatgtcaagtacaacaatgataagagcagagactacactcgacctgacctgccctctggagacagccagccttcactagaccagaccatggcagcagcctttggtgcgcccggcataatgtcagcctctccgtatgcaggagccgggttccctcccacctttgccatccctcaggccgcaggcctctctgtccctaatgtccatggagccttggcccccctggccatcccgtctgctgctgctgctgctgcggccagccgcattgccatcccagggttggcaggtgctgggaatctggccatgagccacctgaacgggcacaagctgcacgggaagtcagtgcgcattacactgtccaagcatcagagtgtgcagctgcctcgggagggtcaggaggaccagggcctgaccaaggactatggcagctccccgctgcaccgcttcaagaaaccaggctccaagaacttccagaacatctttccaccctcagctaccctgcacctctccaacatcccgccctctgtgtcagaggacgacctcaagagcctcttctccagcaacggtggtgtggtcaaaggcttcaagttcttccagaaggaccgcaagatggcactgatccagatgggctctgtggaggaggctgtgcaggcgctgattgaactgcacaaccatgacctgggcgagaaccaccacctgcgagtgtccttttccaagtccaccatctaTCTAGA	1529 nt
pCMV3-SV40-EGFP(2A) Puro-c3×Flag-△*Ptbp1*(478-553)	GGTACCGCCACCatggacggcatcgtcccagacatagcagtcggtacaaagcggggatccgacgagctcttctccacgtgtgtcagcaacggccccttcatcatgagcagctctgcctcagcagccaatggaaacgatagcaagaagttcaaaggtgacaacaggagcgcaggagtcccttccagagtcatccatgtcagaaagctgcccagcgatgtcactgagggcgaggtcatctccctagggctgccctttggaaaggttaccaaccttctcatgctgaaggggaagaaccaggccttcattgagatgaacacagaggaggctgccaacactatggttaactactatacatcggtggcgccagtgcttcgtggacagcccatctacatccagttctccaaccacaaagagctcaagaccgacagctcgcccaaccaggcacgtgcccaggcagccctgcaggctgtaaactccgtccagtctggaaacctggccttggcagcgtccgctgctgccgtggatgcaggaatggcaatggcagggcagagcccagtgctcaggatcattgtggaaaaccttttctacccagtgaccctggacgtgctgcaccagatcttctctaagtttggcaccgtcctgaagatcatcacgttcaccaagaacaaccagttccaggcgctgctgcagtatgctgaccctgtgagcgcccagcatgccaagctgtccctggatggccagaacatctacaacgcctgctgcacgctgcgcatcgacttctccaagctcaccagtctcaatgtcaagtacaacaatgataagagcagagactacactcgacctgacctgccctctggagacagccagccttcactagaccagaccatggcagcagcctttggtgcgcccggcataatgtcagcctctccgtatgcaggagccgggttccctcccacctttgccatccctcaggccgcaggcctctctgtccctaatgtccatggagccttggcccccctggccatcccgtctgctgctgctgctgctgcggccagccgcattgccatcccagggttggcaggtgctgggaattctgtccttttggtcagcaatctgaaccctgagagagtcacaccccaaagcctctttattctcttcggcgtctacggtgatgtgcagcgggtgaagatcctgttcaataagaaggagaacgcacttgtgcagatggcagacggcagccaggcccagctggccatgagccacctgaacgggcacaagctgcacgggaagtcagtgcgcattacactgtccaagcatcagagtgtgcagctgcctcgggagggtcaggaggaccagggcctgaccaaggactatggcagctccccgctgcaccgcttcaagaaaccaggctccaagaacttccagaacatctttccaccctcaaccatctaTCTAGA	1457 nt
