# Supplementary material for: PTBP1-mediated inhibition of circular RNA SCMH1 biogenesis impairs brain recovery after ischemic stroke
Source: Theranostics. 2026 Jan 1;16(1):239–53. doi: 10.7150/thno.114179 (PMC12665102; doi:10.7150/thno.114179)
Supplement: Supplementary file 1 — Supplementary figures. [file thnov16p0239s1.pdf]

Supplemental Figure for

PTBP1-Mediated Inhibition of circSCMH1 Biogenesis Impairs Brain Recovery after Ischemic Stroke

Ying Bai[1†], Bing Han[1,2†], Yi Zhang[1], Liying Wen[1], Chang Liu[1], Yu Wang[1], Jiafang Cui[1], Bingjing Zheng[1], Ningbo Cai[1], Lian Xu[1], Ling Shen[1], Yuan Zhang[1], Honghong Yao[1,3,4]*

[1] Department of Pharmacology, Jiangsu Provincial Key Laboratory of Critical Care Medicine, School of Medicine, Southeast University, Nanjing 210009, China;

[2] Southeast University Affiliated Liangjiang Hospital, Nanjing Pukou People's Hospital, Southeast University, Nanjing 211899, China;

[3] Co-innovation Center of Neuroregeneration, Nantong University, Nantong 226001, China;

[4] Institute of Life Sciences, Key Laboratory of Developmental Genes and Human Disease, Southeast University, Nanjing 210096, China;

†These authors contributed equally to this work

***To whom correspondence should be addressed:**

Honghong Yao, PhD., Department of Pharmacology, Jiangsu Provincial Key Laboratory of Critical Care Medicine, School of Medicine, Southeast University, Nanjing, 210009, Jiangsu, China; Tel: +86 25 8327 2551; E-mail: yaohh@seu.edu.cn.

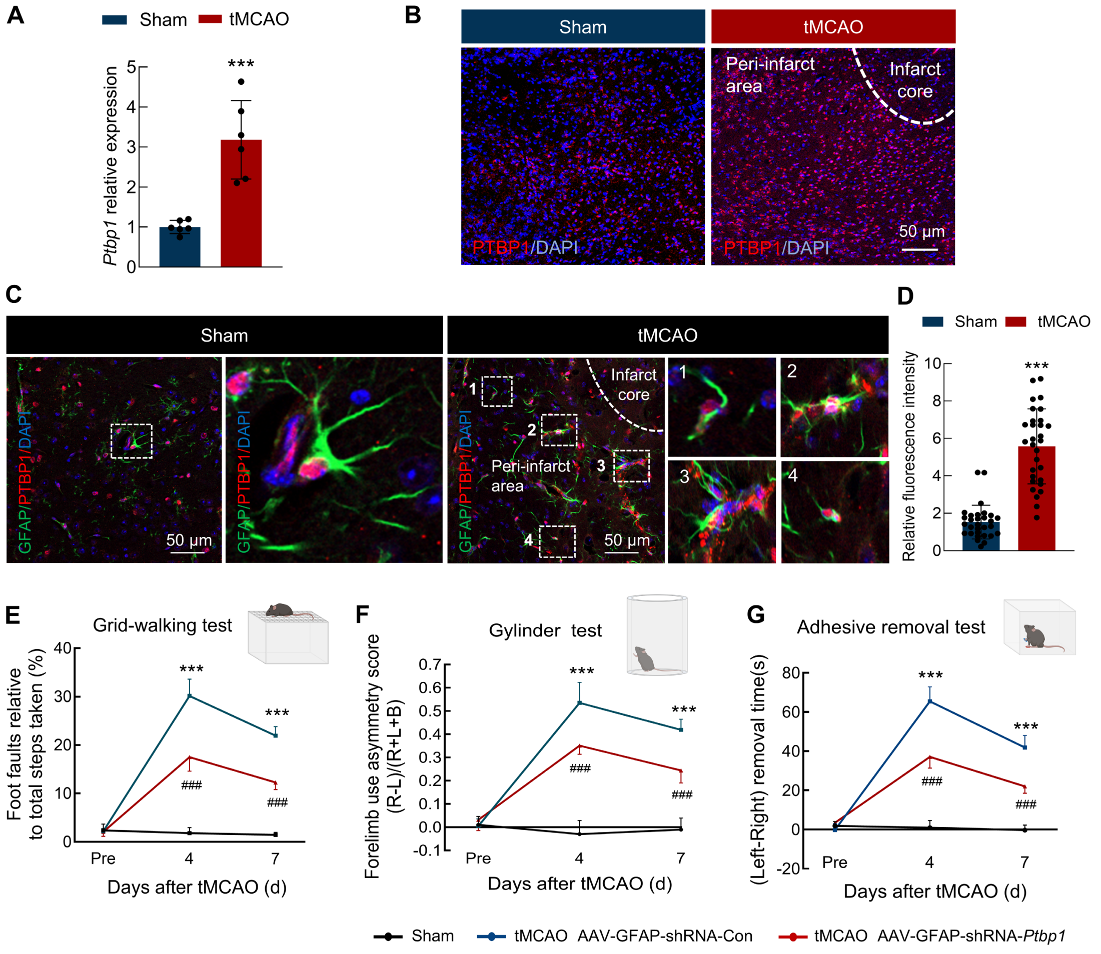

Supplemental Figure 1. PTBP1 functions as an RNA-binding protein for circSCMH1 and significantly increases in the infarct area in tMCAO stroke model. (A) The expression of *Ptbp1* in the peri-infarct area of tMCAO mice compared with that in sham mice by real-time PCR. n=6 samples/group. $^{***}P < 0.001$ versus Sham group. **(B)** The immunofluorescence staining of PTBP1 in the peri-infarct area of tMCAO mice compared with that in sham mice. Scale bar: 50 µm. **(C-D)** The immunofluorescence staining of PTBP1 in astrocytes on the peri-infarct area of tMCAO mice compared with that in sham mice. $^{***}P < 0.001$ versus sham group. **(E-G)** AAV-GFAP-shRNA-*Ptbp1* improved behavioral recovery at different time points after tMCAO mouse stroke model, as measured by the grid walking test **(E)**, cylinder test **(F)**, and adhesive removal test **(G)**. n = 12/group. All data are presented as the mean ± SD. $^{***}P$ <0.001 versus Sham group, $^{\#\#\#}P$ <0.001 versus tMCAO AAV-GFAP-shRNA-Con group.

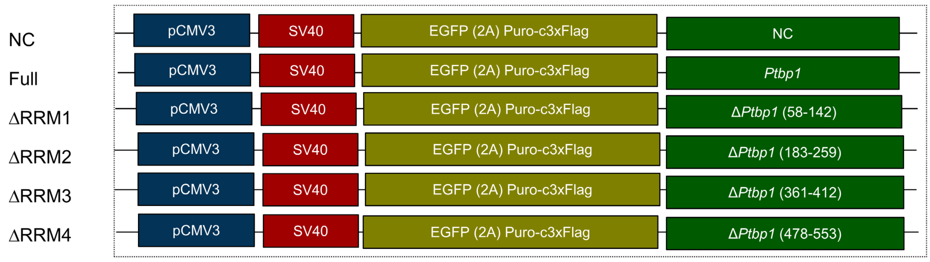

Supplemental Figure 2. Schematic diagram of *Ptbp1* plasmid construction.

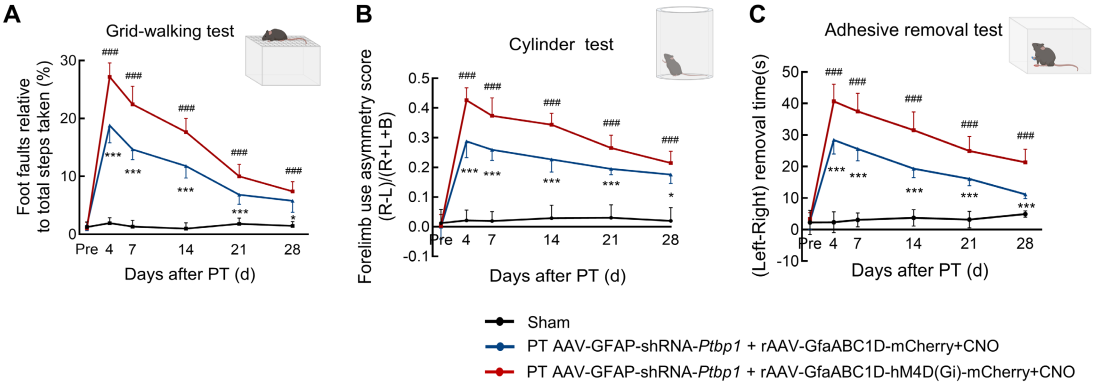

Supplemental Figure 3. Loss of function in astrocytes markedly diminished the therapeutic efficacy of AAV-GFAP-shRNA-*Ptbp1*. (A-C) AAV-GFAP-shRNA-*Ptbp1* improved behavioral recovery at different time points after PT mouse stroke model, as measured by the grid walking test **(A)**, cylinder test **(B)**, and adhesive removal test **(C)**. n = 12/group. All data are presented as the mean ± SD. $^{*}P$ <0.05, $^{***}P$ <0.001 versus Sham group, $^{\#}P$<0.05, $^{\#\#}P$<0.01, $^{\#\#\#}P$<0.001 versus PT AAV-GFAP-shRNA-*Ptbp1*+ rAAV-GfaABC1D-mCherry+CNO.

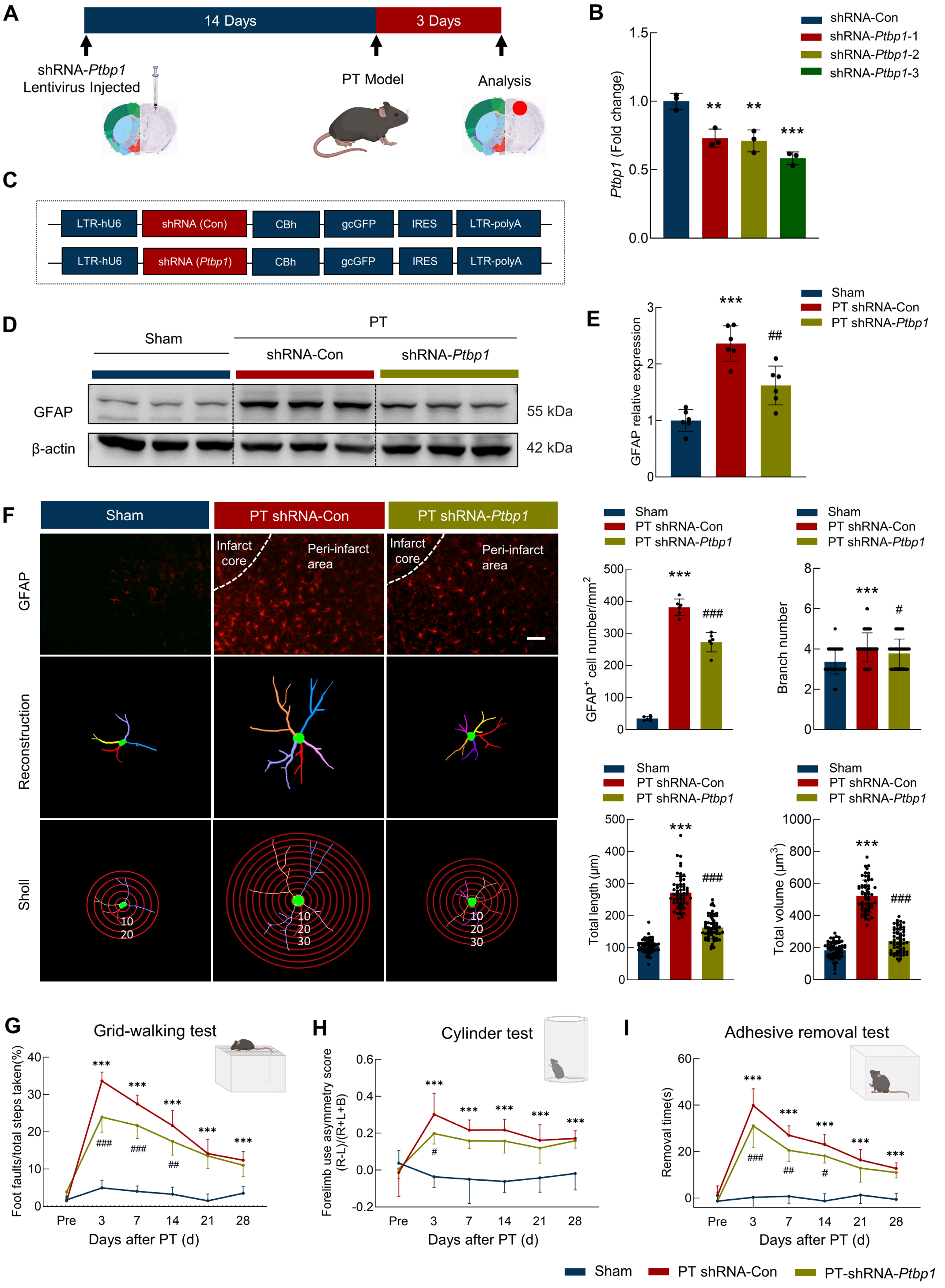

Supplemental Figure 4. Downregulating PTBP1 significantly inhibits the activation of astrocytes and promotes the function recovery after ischemic stroke. (A) Timeline of the experimental procedure in the PT mouse model. **(B)** Expression of *Ptbp1* transduced with shRNA-*Ptbp1*-1/2/3. $^{**}P$ <0.01, $^{***}P$ <0.001 versus shRNA-Con group. **(C)** Schematic of the lentivirus encoding *Ptbp1*. **(D-E)** Western blot analysis of GFAP expression after PT stroke model. Three representative immunoblots from 6 mice/group are presented. $^{***}P$<0.001 versus Sham group. $^{\#\#}P$<0.01 versus PT+shRNA-Con group. **(F)** Effect of PTBP1 on the astrocyte activation. Representative images of astrocyte immunostaining for GFAP in the peri-infarct area, followed by

3D reconstruction and Sholl analysis, branch number, total length, and total volume. 60 cells/group. Scale bar: 50 μm $^{***}P$ <0.001 versus Sham group. $^{\#}P$ <0.05, $^{\#\#\#}P$ <0.001 versus PT+shRNA-Con group. **(G-I)** lentivirus-shRNA-*Ptbp1* improved behavioral recovery at different time points after stroke, as measured by the grid walking test **(G)**, cylinder test **(H)**, and adhesive removal test **(I)**. n = 12/group. All data are presented as the mean±SD. n=6 mice/group, $^{***}P$ <0.001 versus Sham group.

$^{\#}P < 0.05$, $^{\#\#}P < 0.01$, $^{\#\#\#}P < 0.001$ versus PT shRNA-Con group.

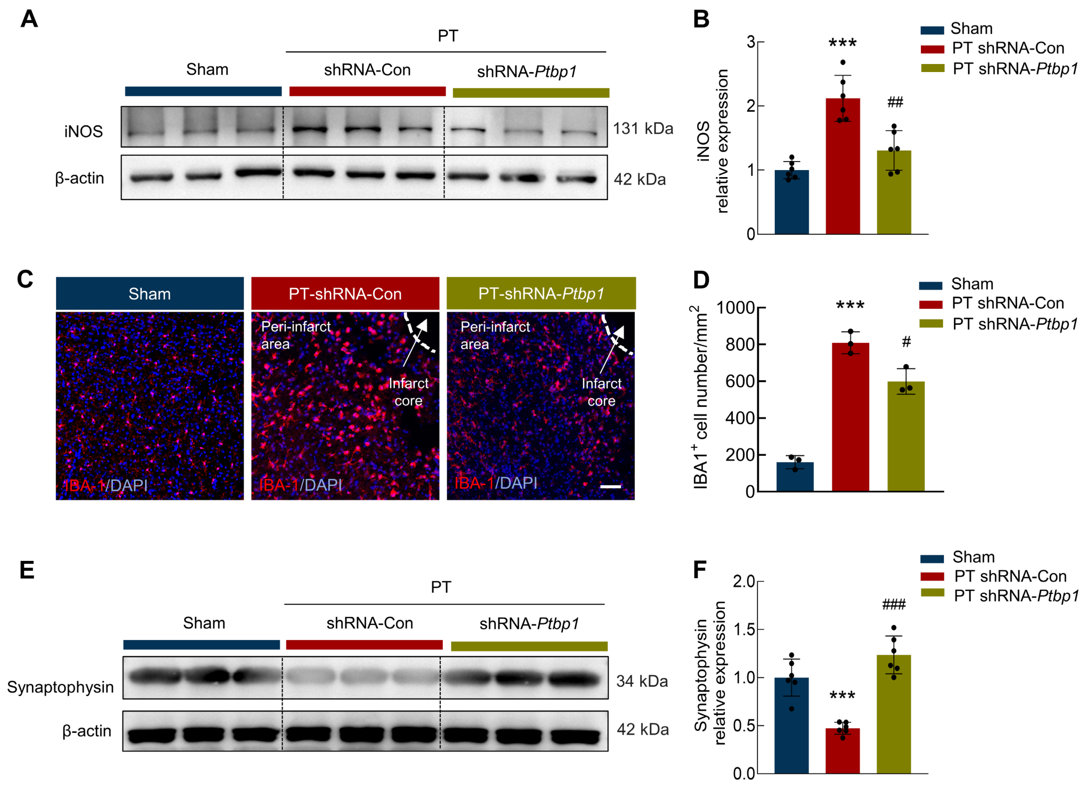

Supplemental Figure 5. Downregulating PTBP1 reduces neuroinflammation and promotes the neuroplasticity. (A-B) Western blot analysis of iNOS expression after PT stroke model. Three representative immunoblots from 6 mice/group are presented. $^{***}P$ <0.001 versus Sham group. $^{\#\#}P$ <0.01 versus PT+shRNA-Con group. **(C-D)** Immunohistochemistry using antibodies against IBA-1. Scale bar: 50 µm. $^{***}P$ <0.001 versus Sham group. $^{\#}P$<0.05 versus PT+shRNA-Con group. **(E-F)** Western blot analysis of synaptophysin expression after PT stroke model. $^{***}P$ <0.001 versus Sham group. $^{\#\#\#}P$ <0.001 versus PT+shRNA-Con group. Three representative immunoblots from 6 mice/group are presented.
